# Multifunctional Magnetic Nanocolloids for Hybrid Solar-Thermoelectric Energy Harvesting

**DOI:** 10.3390/nano11041031

**Published:** 2021-04-18

**Authors:** Elisa Sani, Maria Raffaella Martina, Thomas J. Salez, Sawako Nakamae, Emmanuelle Dubois, Véronique Peyre

**Affiliations:** 1 CNR-INO National Institute of Optics, Largo E. Fermi, 6, I-50125 Firenze, Italy; martinamariella@gmail.com; 2 Service de Physique de l’Etat Condensé, SPEC, CEA, CNRS, Université Paris-Saclay, CEA Saclay, F-91191 Gif-sur-Yvette, France; thom222@free.fr (T.J.S.); sawako.nakamae@cea.fr (S.N.); 3 École des Ponts ParisTech, 6 et 8 Avenue Blaise Pascal, Champs-sur-Marne, F-77455 Marne-la-Vallée, France; 4 Laboratoire Physicochimie des Electrolytes et Nanosystèmes Interfaciaux (PHENIX), CNRS, Sorbonne Université, 4 Place Jussieu, F-75005 Paris, France; emmanuelle.dubois@sorbonne-universite.fr (E.D.); veronique.peyre@sorbonne-universite.fr (V.P.)

**Keywords:** Seebeck effect, direct absorption solar collectors, nanofluids, concentrating solar power, maghemite, thermoelectricity

## Abstract

Present environmental issues force the research to explore radically new concepts in sustainable and renewable energy production. In the present work, a functional fluid consisting of a stable colloidal suspension of maghemite magnetic nanoparticles in water was characterized from the points of view of thermoelectrical and optical properties, to evaluate its potential for direct electricity generation from thermoelectric effect enabled by the absorption of sunlight. These nanoparticles were found to be an excellent solar radiation absorber and simultaneously a thermoelectric power-output enhancer with only a very small volume fraction when the fluid was heated from the top. These findings demonstrate the investigated nanofluid’s high promise as a heat transfer fluid for co-generating heat and power in brand new hybrid flat-plate solar thermal collectors where top-heating geometry is imposed.

## 1. Introduction

In the current pursuit to improve energy conversion, production, and storage efficiency of renewable technologies, hybridization (i.e., combining different energy production technologies in a single system) is considered a promising approach. The advantage is even more relevant if the hybridized energy sources are all renewable and are brought to their highest efficiency by minimizing every energy loss channel in energy transfer and conversion stages. In the case of solar energy, such hybridization efforts are often made by combining photovoltaics (PV) (e.g., silicon, organic cells, etc.), solar thermal collectors (STC), photochemical synthesis, and other renewable energy technologies. For example, the hybridization of PV cells, arguably the most exploited form of solar energy-harvesting technology, with windmills and batteries as well as thermionic and thermoelectric generators, is widely studied.

Thermoelectricity (TE) describes materials’ intrinsic ability to convert heat into electricity and vice versa. A material’s TE energy conversion *capacity* is expressed in terms of the Seebeck coefficient: *Se* = −Δ*V*/Δ*T* where Δ*T* is the applied temperature difference across its body and Δ*V* is the resulting electric potential difference generated in response. Alternatively, the conversion *efficiency of a given TE material* is expressed by a dimensionless parameter named “figure of merit” *ZT*, which combines three transport properties, i.e., the electrical conductivity σ, the thermal conductivity κ, and *Se*: *ZT* = (*σSe*^2^/*κ*)*•T*. Today, solid-state, semiconductor-based thermoelectric generators (TEG) dominate the TE-technology landscape. Due to their low efficiency and the use of rare and toxic elements, however, the application potential of TEGs as a stand-alone renewable energy technology is often said to be bleak [1]. On the other hand, TEGs are showing promising results as a secondary waste-heat recovery tool from a primary energy harvester, such as photovoltaics and solar thermal collectors. Several integration concepts of TEGs in hybrid solar harvesters in combination with the PV [2] and the STCs [3] have been tested since the 1950s [4,5,6]. In these systems, the commercially available TE modules (p- and n-type semiconductor alloys such as SiGe and BiTe or perovskites with the *Se* coefficient in the order of ~100 μV/K and the *ZT* values of 0.5~1) are used, providing device efficiency improvements in the range of 1~5% [7].

In the case of STC, hybridization with a TEG module is particularly advantageous as it enables the co-generation of ‘heat’ and ‘electricity.’ Conventional, low-mid temperature, solar thermal collectors consist of a dark surface devoted to sunlight absorption and to heat exchange with a thermal fluid. These systems are known to suffer from efficiency limitations due to the thermal resistance at the absorber–fluid interface. An improvement was proposed in so-called direct-absorption solar collector (DASC) scheme, whose core is a dark fluid working both as a volumetric light absorber and a heat exchanger. The DASC idea dates back to 1975 [8], using India ink dissolved in water, which, however, was not suitable for practical application due to thermal- and light-induced degradations. Thus, DASCs were almost abandoned for many years, until the development of nanotechnology, which allowed the production of new nanoparticle-laden fluids (nanofluids) with superior stability properties [9,10,11,12,13,14,15]. Stable nanofluids containing magnetic nanoparticles (often called ferrofluids) have recently gained increased interest in different application fields, such as electrical and thermal engineering [16,17,18], medicine and biology [19,20], and sensing and optical devices [21,22]. Recently, a ferrofluid containing Fe_3_O_4_ nanoparticles was tested in a linear parabolic solar collector as a DASC fluid [23]. The authors obtained an efficiency increase with respect to the conventional collector architecture, exploiting both direct sunlight absorption by the ferrofluid and its magnetic-field-enhanced thermal conductivity.

In parallel, large thermoelectric effects were reported in liquid electrolytes including ferrofluids [24,25,26,27]. In general, the *Se* coefficient values of liquid electrolytes are in the range of 1~10 mV/K, an order of magnitude higher than that of semiconductor counterparts. Among multiple TE phenomena occurring in liquid electrolytes, the most robust is that of thermogalvanic effects, i.e., the temperature-dependent electrochemical reactions between the dissolved redox-couple molecules and the electrodes. In the case of charged colloidal suspensions, such as ferrofluids, the thermoelectric diffusion effect of large molecules and particles can be tuned to further boost the *Se* coefficient with only a small particle concentration [28]. The best-performing liquid-thermoelectric generator today is reported to produce maximum power output of 12 W/m^2^ with ferro/ferricyanide redox salts dissolved in water with Δ*T* of ~80 K (without nanoparticles’ inclusions) [29]. The limiting factors of liquid TEGs’ operation are their poor electrical conductivity and the small operation temperature window, both of which are much smaller than those of semiconductor-based TEGs. To this end, the current research trends focus on the use of ionic liquids to increase both the conductivity and the operational temperature limit [30], the synthesis of novel redox-couples and electrolytes with ever higher Seebeck coefficient [31], and the nanostructuration of electrodes to increase their active surface area [32].

Combined, a flat-plate, solar-thermal collector with a very large, heated surface offers an ideal application opportunity for these multifunctional ferrofluids to co-generate heat (through an efficient sunlight absorption) and electricity (through thermoelectric conversion). To assess such co-generation feasibility, here we report the first experimental investigation on the thermoelectric and the optical properties of stable aqueous ferrofluids containing maghemite nanoparticles.

## 2. Concepts

### 2.1. Basic Concepts on the Thermoelectric Energy Conversion in Liquid Electrolytes and Colloidal Suspensions: ‘Thermocell’

A simple thermo-electrochemical cell, or a *thermocell* for short, considered here was filled with a solution composed of a liquid electrolyte, redox couple salts, and, in the case of nanofluids such as ferrofluids, charged (magnetic) nanoparticles. The electric neutrality of the solution was maintained by the presence of free counter-ions in the electrolyte. The two ends of the thermocell were sealed hermetically with conducting electrodes that exchanged electrons with the redox couple to generate electricity. When a temperature gradient was applied across the cell, several thermoelectric phenomena took place, producing a net electric potential difference between the two electrodes. The physical and electrochemical mechanisms behind such phenomena in liquids are quite different from the Seebeck effect in solids. These include the thermogalvanic effect of redox species, the thermodiffusion of ionic species, and the thermo-electrostatic effects (e.g., temperature-dependent formation of electronic double layer or adsorption of charged particles) at the electrode–liquid interfaces [24,33].

#### 2.1.1. Thermogalvanic Effect

The thermogalvanic effect, the most dominant of the three effects, describes the electrochemical reaction potential of dissolved redox species. The oxidant and reductant, denoted here as *A* and *B*, exchange *n*-electrons as

(1) A+ne↔B

The *n*-electrons are either given to or taken from the electrodes, generating an electrical current from the thermocell. The temperature dependence of the redox reaction potential creates a voltage difference (Δ*V*) across the thermocell when a temperature gradient is applied between two electrodes. This temperature dependence is related to the reaction entropy difference, Δ*S_rc_*.

(2) $$-\frac{\Delta V}{\Delta T}=-\frac{\Delta S_{rc}}{e}=Se_{TG}$$

The Δ*S_rc_* is composed of the standard reaction entropy of a given redox couple and the Nernst term, which is a function of temperature, redox molecules’ concentrations, and the activity coefficient. The last depends strongly on the ionic strength of the surrounding solution [34]. The most widely studied type of thermocells is that containing aqueous potassium ferro/ferricyanide redox solutions, presenting *Se* values (*Se_TG_*, subscript *TG* for thermogalvanic) between 1 and 2 mV/K. The highest (published) *Se* coefficient was found in thermocells containing cobalt-based redox couples dissolved in ionic liquids, showing *Se_TG_* > 2 mV/K over a wide temperature range extending well above 100 °C [30].

#### 2.1.2. Thermoelectro-Diffusion Effect

In thermocells containing large-sized, charged species such as colloidal particles and macro-ions or molecules the TE potential production in liquids is further coupled to their movement. It is closely related to the thermodiffusion of charged species, also known as the Soret effect, which describes the concentration (Φ) gradient induced by a temperature gradient. The particle diffusion inside a thermocell is a long and slow process during which the thermoelectro-diffusion-induced electric potential evolves from the initial state (immediately after the application of a temperature and before the concentration gradient is created) to the steady state, i.e., when all the thermodiffusion of all species has reached the Soret equilibrium. The corresponding Seebeck coefficients, $Se_{TED}^{ini}$ and $Se_{TED}^{st}$, are [35]:

(3) $$Se_{TED}^{ini}=\sum_{i}\frac{t_{i}{\hat{S}}_{i}}{\xi_{i}e},\;Se_{TED}^{st}\approx 0$$

where *t_i_*, *ξ_I_,* and *Ŝ_i_* denote the Hittorf number, effective dynamic charge, and the Eastman entropy of transfer of the *i*th charged species (particles, ions, molecules, etc.). In the context of colloidal solutions, *Ŝ_i_* describes the interaction between the particle surface and its surrounding liquid (see [36,37] for the exact definition of *Ŝ* as employed here). The Hittorf number (see, for example, [38]) is the fractional ionic conductivity of a given charged specie, *σ_i_*, with respect to the total conductivity *σ_tot_*, i.e.,

(4) $$t_{i}=\frac{\sigma_{i}}{\sigma_{tot}}=\frac{z_{i}\xi_{i}e^{2}n_{i}D_{i}}{\sum_{i}z_{i}\xi_{i}e^{2}n_{i}D_{i}}$$

where *z_i_*, *n_i_*, and *D_i_* are the valence charge (or static effective charge), number concentration, and the diffusion coefficient. The $Se_{TED}^{ini}$ stemming from the redox species and other electrolyte ions is quite small in comparison to the thermogalvanic term, *Se_TG_*, and thus it is customary to ignore this term. On the contrary, in a thermocell containing charged colloidal particles with large *ξ_I_* and *Ŝ_i_* values, both TED and TG terms contribute to the total *Se* coefficient, i.e.,

(5) $${\mathrm{Se}}^{\mathrm{ini}}={\mathrm{Se}}_{\mathrm{TG}}+Se_{TED}^{ini}=-\frac{\Delta S_{rc}}{e}+\sum_{i}\frac{t_{i}{\hat{S}}_{i}}{\xi_{i}e}$$

Once the Soret equilibrium is reached, the *thermoelectro*-diffusion contribution to the *Se* coefficient (measurable at the electrodes) disappears due to the rearrangement of the charged species, screening the electrodes from any internal electric field in the bulk of the solution [35,39] and thus

$${\mathrm{Se}}^{\mathrm{st}}={\mathrm{Se}}_{\mathrm{TG}}=-\frac{\Delta S_{rc}}{e}$$

Note that in the present work where a thermal gradient spanned a length of ~ cm of our experimental thermocell, the Soret equilibrium of nanoparticles was reached after tens of hours (50–80 h), much longer than a typical experimental time scale. Therefore, all experimental *Se* values presented hereafter correspond to $Se_{TED}^{ini}$.

## 3. Experimental

### 3.1. Sample Preparation and Characterization

The ferrofluids studied here were composed of maghemite (γ-Fe_2_O_3_) nanoparticles (NPs) coated with a statistical co-polymer made of equimolar acrylic and maleic acid monomers (PAAMA), of average molecular weight 3000 g/mol, dispersed in water. These coatings gave large structural charges to the nanoparticles (20 elementary charges/nm^2^) and ensured the ferrofluid’s colloidal stability. The PAAMA remained strongly bound to the surface without any equilibrium with free PAAMA in solution [40]. The sample ferrofluids were synthesized initially from an acidic aqueous ferrofluid (HNO_3_, pH =1.8, with a NP volume fraction φ = 2% vol.) obtained via the ‘Massart’ method, followed by size sorting when required [41,42]. Two acidic FFs (hereafter denoted FF1, used for thermoelectric investigation, and FF2, for the optical measurements) had different size distributions. In FF1, the log-normal distribution parameters were d_0_ = 7.2 nm and σ = 0.17 (from the room temperature magnetization measurements [43]), whereas in FF2, d_0_ = 6.0 nm and σ = 0.43 (determined by SAXS). The size sorting was crucial for the thermoelectric data analysis because of the *Se* coefficient’s dependence on the thermodiffusive property of charged nanoparticles (Equation (4)) and, thus, on their average diffusion coefficient, D. The latter, on its turn, depended on the NP size. The optical absorption spectrum will depend both on the nanoparticle concentration (volume fraction) and, in principle, on the size distribution as well. However, as discussed in Section 4.3, the dependence was much stronger on the concentration and, thus, no further sorting step was involved.

The PAAMA ligand coating on the nanoparticle surface was then introduced via three successive steps according to the “precipitation-redispersion procedure”, as described in [44,45]. The resulting ferrofluid consisted of negatively charged γ-Fe_2_O_3_ nanoparticles coated with PAAMA (13.2 ± 1.9 monomers of acrylic/maleic acid per nm^2^ [40]), resulting in the structural charge of −3300 e. The electrical neutrality was ensured by ammonium ions (counter-ions). The final pH was around 9.2. After dilution with water (18 MΩ), the volume fractions of nanoparticles were 0.54% vol. and ~0.05% vol. for FF1 and FF2, respectively.

### 3.2. Thermoelectric and Electrical Characterizations

The Seebeck coefficient (*Se*), the power-output (P), and the AC ionic conductivity (*σ*) of FF1 were investigated as a function of NP volume fraction (Φ, between 0 and 0.5% vol., and temperature, using a homemade cylindrical thermocell) [25,28,35]. The sample liquid volume was ~170 μL (6 mm in diameter and 6 mm in height, cf., Supplementary Materials Section S4), which was hermetically sealed at the top and bottom by Pt electrodes (thin disk, 99.99% purity, AlfaAesar, Haverhill, MA, USA) pressed against the cell body by copper blocks. Before each measurement, the electrode surface was cleaned with concentrated HCl (Sigma-Aldrich, St. Louis, MO, USA, 37%wt.) and washed by ultrasonication in deionized water. As a redox couple, ferri/ferrocyanide (Fe(CN)_6_^−3^/Fe(CN)_6_^−4^) pair was used, introduced in the form of (K_3_Fe(CN)_6_ (Sigma Aldrich, 99,98% purity) and K_4_Fe(CN)_6_ (Sigma Aldrich, 99,95% purity). The redox concentration was 3 mM each for *Se* coefficient and AC conductivity measurements, while both 3 mM and 400 mM were used in the power-output measurements (see Results section for explanation).

The AC ionic conductivity measurements were performed in isothermal conditions (at T_mean_ = 25, 35 and 45 °C) using a precision LCR meter (HP 4284A) at 100 kHz, at which the out-of-phase component of the impedance became null. The *Se* coefficient was determined from the open-circuit voltage ΔV between the top and bottom electrodes in non-isothermal conditions (T_mean_ = 25, 35, and 45 °C with ΔT = 10 K between the two electrodes), measured using a high-impedance electrometer (Keithley 6514, 10^14^ Ω input resistance, Keithley, Solon, OH, USA). The total *Se* coefficient was then calculated from a simple relation: *Se* = −Δ*V*/Δ*T* in the open-circuit condition. The thermoelectric power-output measurements were carried out with a Δ*T* of 30 K (20–50 °C, T_mean_ = 35 °C) in a closed-circuit configuration, i.e., the thermocell was connected in parallel to a variable-range discharge resistor (10 Ω to 10 MΩ). More detailed measured schemes are given in the Supplementary Materials Section S4. For the *Se* measurements, the thermocell was always heated from the top in order to avoid the natural convection within the fluid. For the power measurements, both heating directions (bottom and top heating) were used to examine the effect of convection on the thermocell’s power output.

### 3.3. Optical Characterization

The optical transmittance of sample was measured using a double-beam, UV-VIS spectrophotometer (PerkinElmer Lambda900, PerkinElmer Waltham, MA, USA), holding the sample in a variable length cell [46,47]. Once obtained, the spectral extinction coefficient *μ*(*λ*) from transmittance measurements, the extinction fraction (EF), of the incident sunlight *I*(*λ*) [48] as a function of the light propagation path length × within the nanofluid was calculated as [13,14]:

(6) $$EF\left(x\right)=1-\frac{\int_{\lambda_{min}}^{\lambda_{MAX}}I\left(\lambda \right)\Delta e^{-\mu \left(\lambda \right)x}d\lambda }{\int_{\lambda_{min}}^{\lambda_{MAX}}I\left(\lambda \right)d\lambda }$$

where the considered integration boundaries (*λ_min_*, *λ_MAX_*) were 300 and 2500 nm.

## 4. Results and Discussion

### 4.1. Ionic Conductivity and Determination of Effective Dynamic Charge

**Figure 1:**
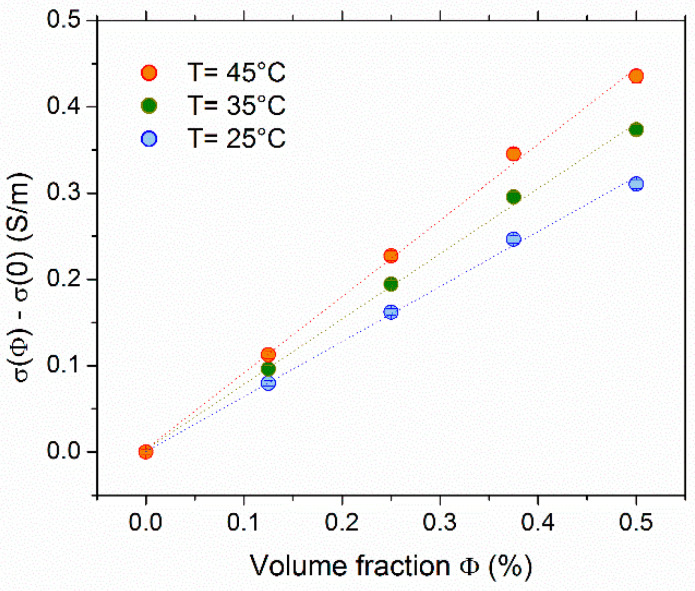
Ionic conductivity (*σ*) of FF1 as a function of the NP volume fraction Φ. The conductivity at Φ = 0, i.e., the contribution from free ions and redox couple ions was subtracted, showing only the contributions from the NPs and the counter-ions surrounding the negatively charged particles (for electro-neutrality).

The ionic conductivity dependency on the NP volume fraction (Φ) in FF1 examined at 25 °C, 35 °C, and 45 °C is shown in Figure 1. The *σ*(Φ) increased with the addition of NPs at all examined temperatures. The conductivity of the base fluid (no NPs but with 3 mM each of redox salts) at 35 °C was determined to be *σ*(0) = 0.312 S/m, corresponding to the thermocell’s ohmic resistance of ~700 Ω.

Within the low concentration range explored, the conductivity was found to be approximately a linear function of NP volume fraction. In the case of negligibly small inter-particle interactions (see Supplementary Materials for justifications), the increase, Δ*σ*(*φ*) = *σ*(*φ*) *−* *σ*(0), was due to the diffusion of charged NPs and the surrounding counter-ions, and it can be expressed as:

(7) $$\Delta \sigma \left(\Phi \right)=\frac{D_{NP}\cdot e^{2}\cdot {\left(\xi_{0}\right)}^{2}\cdot \Phi }{v_{NP}\cdot k_{B}\cdot T}+\frac{D_{ci}\cdot e^{2}.z_{ci}^{2}\cdot \left|\xi_{0}\right|\cdot \Phi }{v_{NP}\cdot k_{B}\cdot T}$$

where *ξ*_0_ is the NP’s dynamic charge (at infinite dilution limit), *vX_NP_* is the individual nanoparticle volume, and *D_ci_* and *z_ci_* are the diffusion coefficient and the valence of number of the counter-ions (in this case, NH_4_^+^). Using *z_ci_* = +1 and *D_ci_* = 1.96 × 10^−9^ m^2^/s at 25 °C [49], *ξ*_0_ = −280 was deduced as the NP’s average dynamic charge. We note that this value is an order of magnitude lower than the bespoke structural charge number due to the large number of counter-ions condensing on the particle surface.

### 4.2. Thermoelectric Properties

#### 4.2.1. Seebeck Coefficient

The initial Seebeck coefficient of FF1 was measured as a function of Φ (in the presence of 3 mM of the redox couple ferri/ferrocyanide) at average temperatures of 25 °C, 35 °C, and 45 °C with a constant temperature difference of ∆*T* = 10 K between the hot and cold electrodes. The measurements were stable and highly reproducible over several weeks. In the absence of nanoparticles, *Se^ini^*(0) was approximately 1.7 mV/K, in agreement with typically reported range of values, 1–1.8 mV/K for Fe(CN)_6_^−3^/Fe(CN)_6_^−4^ redox couple in aqueous media with different ionic strengths [32,50,51,52,53,54,55,56].

**Figure 2:**
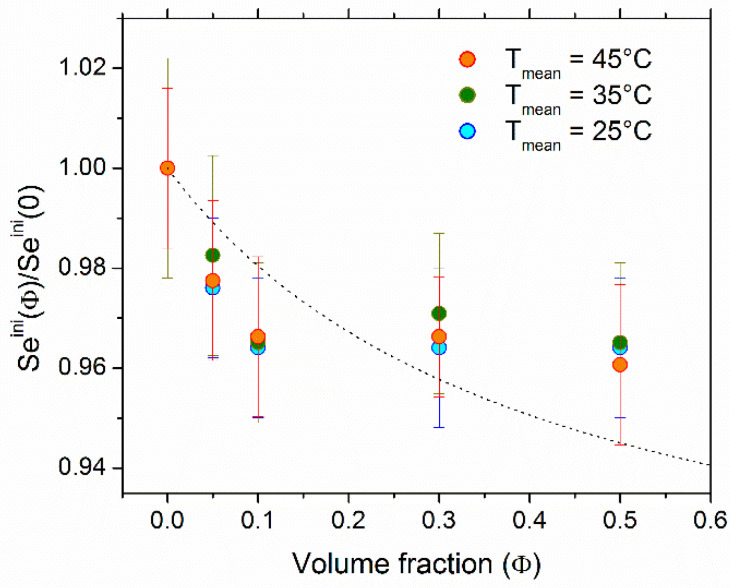
The initial *Se* coefficient normalized to *Se^ini^*(0) as a function of NP volume fraction at mean cell temperature of 25 °C, 35 °C, and 45 °C (*T_mean_* = (*T_hot_* + *T_cold_*)/2). The 3 mM each of ferro/ferricyanide redox couple was present. The Δ*T* = 10 K was used for all measurements. The theoretical curve was produced using Equations (4) and (5) with DNP = 1.80 × 10^−11^ m^2^/s (see Supplementary Materials).

The normalized $Se^{ini}$ as a function of NP concentration at the cell mean temperature T_mean_ = 25 °C, 35 °C, and 45 °C is shown in Figure 2. $Se^{ini}\left(\Phi \right)$ behaved similarly at all three temperatures, i.e., the addition of NPs resulted in a slight reduction of the *Se^ini^* coefficient by approximately 4% at Φ = 0.5% vol.

Considering that the variation of the redox reaction entropy term, Δ*S_rc_*, in Equation (5) to be negligible (Δ*S_rc_* change is induced by the change in the activity coefficient of the redox couple species between the two electrodes due to the addition of nanoparticles and their surrounding counter-ions. We have calculated this effect using tabulated activity coefficient data [49] to be less than 1 µV/K), the dependence of *Se^ini^* (Φ) stems from the *thermoelectro*-diffusion of charged species (NPs and their surrounding counter-ions) whose concentration varies with Φ. Using the effective dynamic charge number (*ξ_NP_* = −280, see Section 4.1) and the diffusion coefficient (*D_NP_* = 1.8 × 10^−11^ m^2^/s, see Supplementary Materials), we calculated the nanoparticles’ Eastman entropy of transfer to be *Ŝ_NP_* = 76 ± 17 meV/K. This value was five times larger than that found for ionically stabilized maghemite nanoparticles (7.6 nm in diameter) in water, *Ŝ_NP_* = 14 meV/K [28], reflecting the effect of large coating molecules on the particle surface.

#### 4.2.2. Power Output

##### Low Redox Concentration, No-Convection Regime

The effect of NP addition on the thermocell’s electrical power production was studied at Φ = 0 and 0.5% vol., first with 3 mM of ferro/ferricyanide redox couple in the “hot over cold” configuration, i.e., no natural convection. *T_mean_* = 35 °C and ∆*T* = 30 K were used throughout the power measurements.

**Figure 3:**
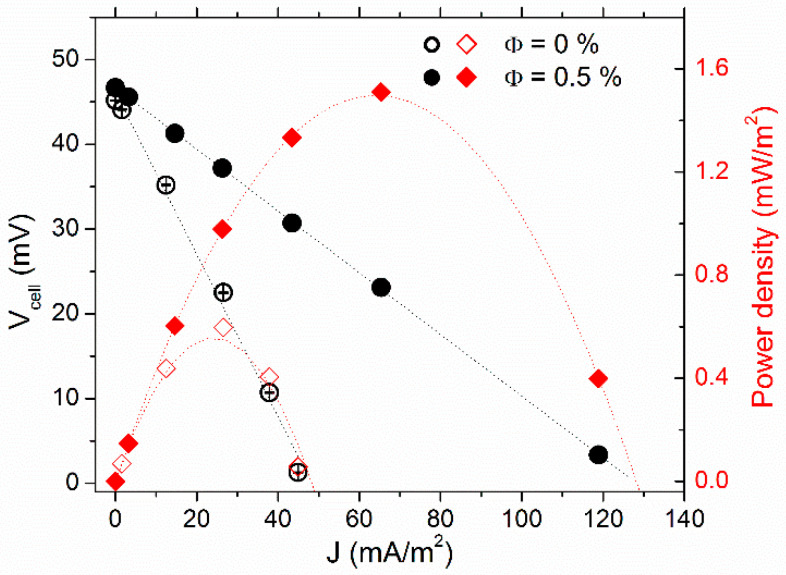
Current density voltage (left *y*-axis) and output power–density curves (right *y*-axis) of FF1 sample with 3 mM each of redox salts at 0% vol. (open symbols) and 0.5% vol. (solid symbols). The measurements were conducted at *T_mean_* = 35 °C with *T* = 30 K, heated from the top.

The thermocell voltage (*V_cell_*) vs. current–density (*J*) curves are depicted in Figure 3, along with the cell’s power-output density (*P*). The *V_cell_* was at its highest in the open-circuit configuration (*J* = 0). As the variable resistance load value (*R_load_*) connected in parallel to the cell (closed-circuit configuration, see Supplementary Materials for more detail) was reduced, *V_cell_* diminished, while the corresponding current, *V_cell_*/*R_load_*, increased. The *V_cell_-J* dependency was almost linear. Thus, one can determine the experimental internal resistances (*R_i_*) of the thermocell from the slope value accordingly: *dV_cell_*/*dJ = R_i_·A*, where *A* is the electrode surface area. *R_i_* was reduced from 34 ± 1 kΩ at Φ = 0% by a factor of 2.6 to 12.9 ± 0.1 kΩ at Φ = 0.5%, nearly the maximum power output density *P_max_* = *J*. *V* increased proportionally (see red curves in Figure 3).

To understand the mechanisms leading to the observed increase in *P_max_* due to the inclusion of NPs, we considered different constituents of the thermocell’s internal resistance, i.e., the ohmic resistance of the fluid, *R_O_*, the mass transfer resistance, *R_MT_*, and the charge transfer resistance, *R_CT_* (Table 1). The latter was less than 1 Ω and was safely neglected. *R_O_* of the FF1 sample was obtained via AC ionic conductivity measurements. At the mean cell temperature of 35 °C, the *R_O_* values were 700 and 300 Ω at Φ = 0 and 0.5% vol., respectively (cf., Section 4.1), nearly two orders of magnitude smaller than the measured *R_i_*. These considerations indicate that thermocell’s internal resistance was dominated by *R_MT_*, whose theoretically expected value corresponding to 3 mM each of ferro/ferri-cyanide redox-couple salt ions (dissolved K^+^ ions included) was ~27 kΩ (see Supplementary Materials Section S5), in the same order of magnitude as the observed value of *R_i_* (Φ = 0). As the redox couple concentration was kept constant in both measurements, the inclusion of NPs served to somehow *accelerate* the mass transfer process of the redox species in the “hot-over-cold” configuration (no-convection regime). The possible explanations for this apparent mass-transfer acceleration are discussed later in the section.

##### High Redox Couple Concentration, No-Convection Regime

Intrigued and encouraged by the significant decrease in the internal resistance (and the resulting increase in the thermocell power output) described above, we examined the thermocell power output using 0.4 M each of ferro/ferricyanide redox couple, with and without NPs. The ohmic resistance of solutions with and without magnetic NPs at *T* = 40 °C was *R_O_* = 9.75 ± 0.5 Ω for both, which is two orders of magnitude lower than that obtained with 3 mM redox salt concentration reported above. In such highly conducting electrolytes, the influence of NPs and their surrounding counter-ions is negligible on the ohmic resistance.

**Figure 4:**
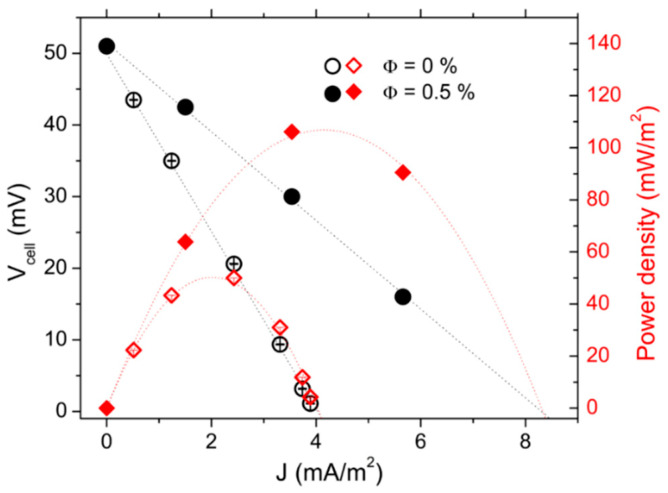
Comparison of voltage-current (circular symbols, left *y* axis) and power density-current (diamond symbols, right *y* axis) characteristics between Φ = 0 and 0.5% vol. in aqueous ferrofluid with 400 mM redox couple. Hot-over-cold configuration.

The power-output measurements were first performed in “hot-over-cold” (no convection) configurations at *T_mean_* = 40 °C and ∆*T* = 40 K. The internal resistance values *R_i_*(Φ) deduced from the *J-V* curves (Figure 4) were *R_i_* (0) = 438± 6 Ω and *R_i_* (0.5%) = 219 ± 5 Ω. Two observations were made. First, these values were much smaller than that obtained with 3 mM each of redox couple salts, but were still larger than the *R_O_* by a factor of ~50 (see above), attesting to the dominance of the mass transfer resistance (*R_MT_*) in the thermocell’s total *R_i_* even at such a high redox concentration. Second, the presence of NPs again caused a marked reduction of *Ri* (and *R_MT_*) by a factor of 2. The resulting increase in the maximum power output was from *P_max_*(0) = 55 mW/m^2^ to *P_max_*(Φ = 0.5%) = 110 mW/m^2^ (Figure 4), similar to the behavior observed in the ferrofluids with low redox concentration, while the overall intensity of the power output was nearly two orders of magnitude larger, reflecting the high redox salt concentration.

**Figure 5:**
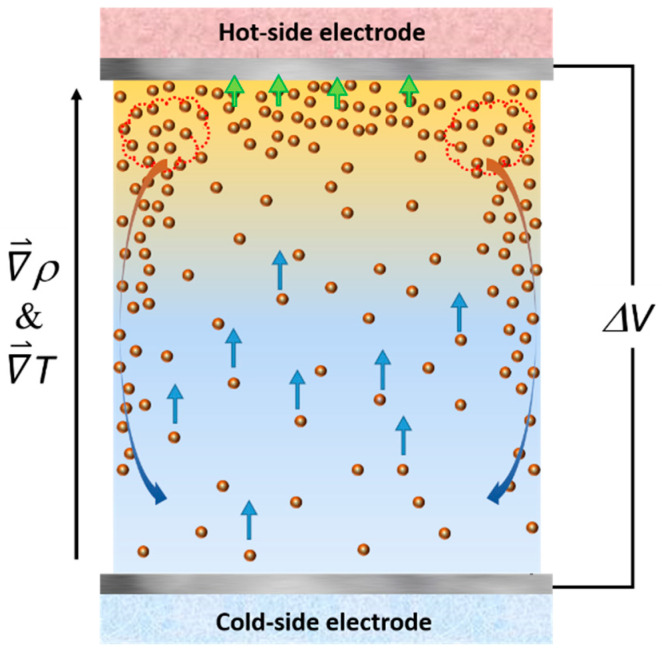
Schematic image of a proposed mechanism creating a convection motion of ferrofluid inside a thermocell. Colored arrows describe different forces experienced by the nanoparticles and the ferrofluid. Blue: thermo-electro diffusion (Soret effect of charged colloidal particles). Green: electrostatic attraction. Orange/Blue: gravitational force acting on a dense layer of ferrofluid.

The origin of the observed *R_MT_* reduction, i.e., the accelerated diffusion of the redox ions in the presence of nanoparticles was not clear. A useful insight was found in the Grätzel solar cells where an increase in the electrical current output was found upon the addition of nano-sized objects such as carbon nanotubes. For example, Chang and co-authors [58] demonstrated that by adding 0.5%wt. of multi-wall carbon nanotubes (MWCNT) to an iodide-based Grätzel solar cell can lead to a 3-fold increase in the short-circuit current and, thus, the power output of the solar cell. Vahlman and co-authors [59] also reported a similar phenomenon in a Grätzel cell containing an ionic liquid (I^−^/I^3−^ redox couple) mixed with black carbon, and demonstrated how the classical liquid diffusion models failed to reproduce the observed current/power increase. In thermogalvanic cells, Salazar and co-authors showed that the mixing of MWCNTs up to 0.6%wt. in ionic liquids (EMI-TFSI and PMI-I (1-Ethyl-3-methylimidazolium bis(trifluoromethylsulfonyl)imide (EMI-TFSI) and 1-propyl-3-methyl imidazolium iodide (PMI-I)) significantly increased the electrical conductivity of the cell, but reduced the *Se* coefficient [60,61]. The overall power output of the cell was determined by the trade-off between the current gain and the voltage loss (*Se* coefficient), both of which depend on the ionic liquid and the redox-couple types. For EMI-TFSI with Co^2+^/Co^3+^ redox couple, the loss in *Se* exceeded the current gain and, thus, the MWCNT addition resulted in an overall decrease of *P_max_*. Conversely, in the PMI-I system with iodine-redox couple, the current gain prevailed, leading to a net *P_max_* increase of about 30% at 0.1%wt. concentration of MWCNT, qualitatively analogous to the power increase/*Se* reduction observed in this study. The authors proposed several rational explanations including a formation of percolated networks of MWCNTs, the interfacial polarization of nanotubes, and the dissociation of ion pairs (of the ionic liquid components). Unfortunately, these mechanisms were not directly applicable to the present study due to some intrinsic differences in the nature of liquid systems investigated, *namely, conducting* MWCNTs vs. *insulating* γ-Fe_2_O_3_ NPs and ionic liquids (*pure salts*) vs. *dilute electrolytes* in water. An alternative hypothesis here was that of the convective motion generation linked to the thermophilic diffusion (thermodiffusion of particles toward the hot region) [62] and the electrostatic adsorption [63,64] of magnetic NPs. Both effects contributed to creating a higher NP concentration near the top (hot) electrode compared to the rest of the thermocell volume. The difference in the density of nanoparticles ($\rho_{NP}$ = 4.88 g/cm^3^) and of water ($\rho_{H_{2}O}$ = 1 g/cm^3^) can cause the FF density in the top layer to be much greater, sufficient for inducing a natural convection-like fluid movement despite its local temperature being higher than the liquid volume below, as illustrated in Figure 5.

The order of magnitude of the concentration increase in the top (hot) FF layer needed to induce such a natural convection motion could be estimated. Assuming that the thermophysical properties of the fluid were unaffected by the inclusion of nanoparticles at these small concentrations, the relative density difference resulting from Δ*T* of 10 K is:

(8) $$\frac{\Delta \rho_{H_{2}O}}{\rho_{H_{2}O}}=10.\;\beta =-3.86\times 10^{-3}$$

where *β* is the isothermal expansion coefficient of water. This density decrease in water can be easily overcome by a small and positive increase in NP concentration ∆Φ to possibly induce convective movement of in the fluid.

(9) $$\Delta \Phi ={\left(1-\frac{\rho_{NP}}{\rho_{H_{2}O}}\cdot \left(\frac{\left(\rho_{NP}-\rho_{H_{2}O}\right)\cdot \rho_{H_{2}O}}{\Delta \rho_{H_{2}O}}+1\right)\right)}^{-1}≅0.02\;\%\;\mathrm{vol}.$$

The estimated value of the NP concentration increase was quite plausible considering the average nanoparticle concentrations of 0.5% used here. In order to test the hypothesis, it will be instructive to measure the thermal transfer coefficient, *h*, of the thermocell in both heat configurations from which one can determine the Nusselt number, *Nu* = *hL*/κ where κ is the thermal conductivity of the fluid and *L* is the cell length. The *Nu* dependency on the nanoparticle concentration will then reveal if the latter indeed enhanced convective motion in nanofluids, as it was demonstrated by Joubert et al. [65] in a ferrofluid similar to ours (SDS-coated Fe_2_O_3_ nanoparticles (15–20 nm in diameter) in water).

##### High Redox Couple Concentration, Convective Regime

**Figure 6:**
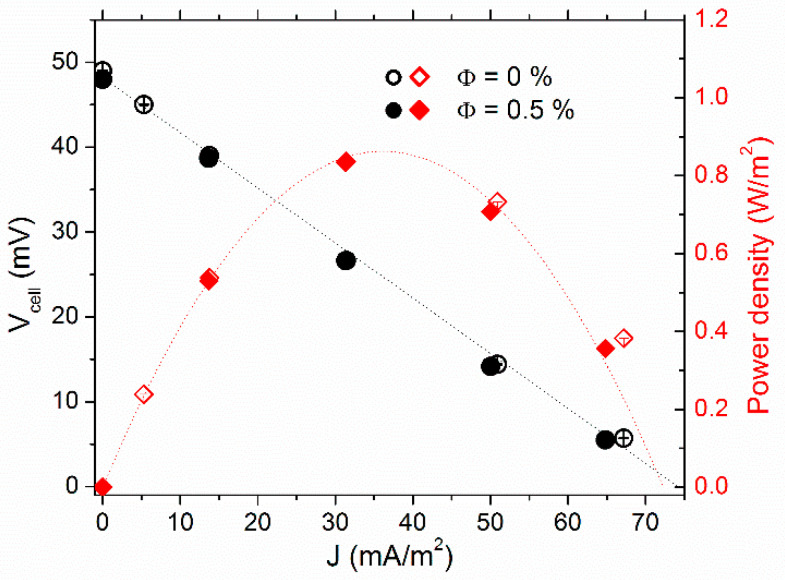
Voltage-current (circle symbols, left *y* axis) and power density-current (diamond symbols, right *y* axis) curve comparison between Φ = 0 and 0.5% vol. in aqueous ferrofluid with 400 mM redox couple. Cold-over-hot configuration.

Lastly, we examined the thermocell’s power output in a “cold-over-hot” configuration to intentionally induce convection (Figure 6). Fluid motions (convection, forced flows) are known to enhance the mass transfer rate of the redox couples (i.e., reduce R_TM_) and, thus, have a positive impact on the power output [66].

As expected, the internal resistance/power output was found to decrease/increase considerably (by a factor of 10) to *R_i_* = 23 ± 1Ω and *P_max_* = 900 mW/m^2^ compared to the “hot-over-cold” configuration. *R_i_* is now in the same order of magnitude of the fluid’s *R_O_*, confirming the benefit of the fluid motion on the redox transport in liquid thermoelectric cells [66]. The effect of the NP inclusion, on the other hand, became insignificant against the convective motion of the fluids, i.e., (i) the convection-induced reduction in *R_MT_* overrode all other effects and/or (ii) the non-uniform spatial distribution of NPs inside the fluid volume as described in the previous section was destroyed by convection.

Combined, one can conclude that the use of magnetic NPs (or any charged colloidal particles, in general) in thermocells becomes advantageous where the “hot-over-cold” configuration is imposed, such as the flat-plate solar collectors. As an extension of this idea, one can consider parabolic trough-type or linear Fresnel-type solar thermal collector to heat a dark nanofluid from the bottom [67] for enhancing the power output via convection, although, in this case, the electrode surface area will become inevitably small.

Lastly, one needs to consider the long-term stability of the ferrofluids. Without the presence of redox salts, the colloidal stability of PAAMA-coated maghemite nanoparticles in aqueous media is verified over several years’ time. The introduction of ferri/ferrocyanide redox couple can disturb such stability. Furthermore, ferricyanide and ferrocyanide complexes become unstable over time and precipitate as coordination polymers related to Prussian Blue (ferric hexacyanoferrate) (see, for example, [68] and references therein). Any change in the nanofluid composition due to undesired chemical reactions between its ionic species results in the change of thermoelectric voltage and power-output behavior. For this reason, we can be sure that such reactions did not take place or remained insignificant (undetectable) over the duration of the measurements described in the present work, i.e., for several weeks. The long-term stability of both redox-couple salts and the colloidal stability of nanoparticles will be addressed in further studies.

### 4.3. Optical Properties

**Figure 7:**
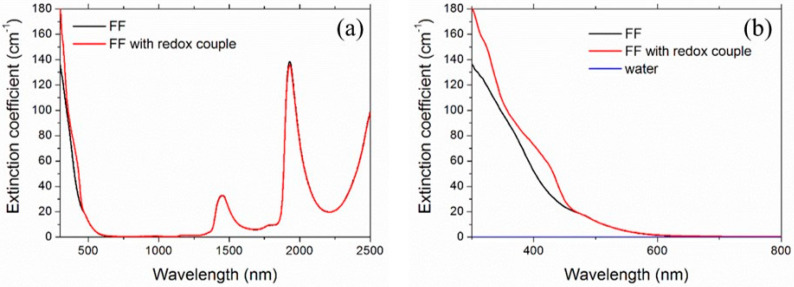
Spectral extinction coefficient of ferrofluids. The plot in (**b**), which is the enlargement of (**a**) in the the range 300–800 nm, also shows the extinction coefficient of water for comparison (blue line practically superimposed to the abscissa axis).

Figure 7 shows the experimental spectral extinction coefficient of the ferrofluid FF2 with and without the redox couple.

The extinction coefficient of a colloidal suspension includes both the optical scattering and the optical absorption contribution. Following the notation in Ref. [69], the extinction and scattering efficiencies are given by:

(10) Qext=4xIm{m2−1m2+2[1+x215(m2−1m2+2)m4+27m2+382m2+3]}+83x4Re{(m2−1m2+2)2}

(11) $$Q_{sca}=\frac{8}{3}x^{4}{\left|\frac{m^{2}-1}{m^{2}+2}\right|}^{2}$$

where *m* is the complex relative refractive index

(12) $$m=\frac{n_{p}+ik_{p}}{n_{f}+ik_{f}}$$

defined in terms of the real (*n_p_*, *n_f_*) and imaginary parts (*k_p_*, *k_f_*) of the complex refractive indexes of particles and fluid, respectively, and *x* is the particle size parameter:

(13) $$x=\frac{2\pi n_{f}a}{\lambda }$$

with *a* being the particle radius and λ the light wavelength in vacuum. In the Rayleigh regime |*m*|*x*<<1, the expression in the square bracket in Equation (10) is approximately unity. The extinction efficiency thus becomes:

(14) Qext=4xIm{m2−1m2+2}+83x4Re{(m2−1m2+2)2}

The relative contribution of the scattering within the overall light extinction phenomenon in the colloid is quantitatively evaluated by defining the scattering albedo as the ratio between the scattering and the extinction efficiencies [69]:

(15) $$\omega =\frac{Q_{sca}}{Q_{ext}}$$

**Figure 8:**
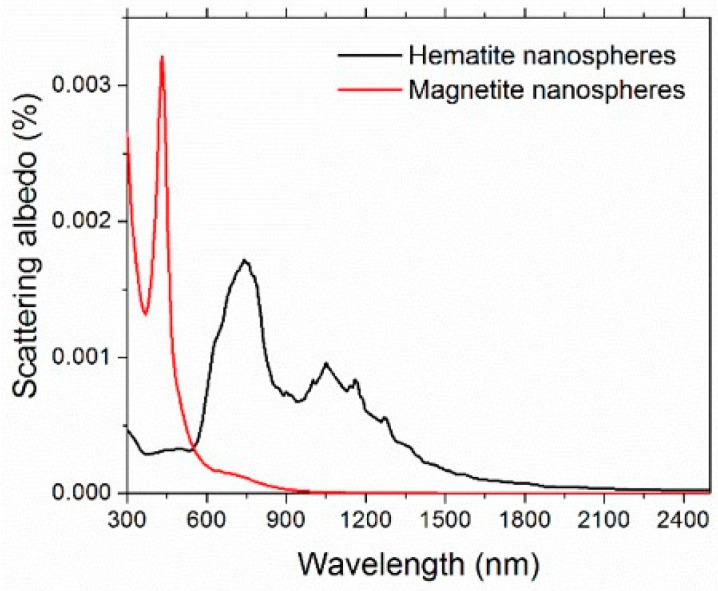
Calculated spectral scattering albedo for 6-nm diameter nanospheres.

Maghemite (γ-Fe_2_O_3_) is one of the most important iron oxide polymorphs, with intermediate characteristics between hematite (α-Fe_2_O_3_) and magnetite (Fe_3_O_4_) [70]. Unfortunately, the complex refractive index of maghemite is not available in the literature. Therefore, to give an estimation of the scattering albedo, we considered both hematite and magnetite [71]. The refractive index of water was taken from [72]. Figure 8 shows the spectral scattering albedo, calculated from Equation (15), considering homogeneous spheres of 6-nm diameter and the 0.05% vol concentration. It is worth noticing that the actual size distribution of FF2 (and FF1, used for thermoelectrical characterization) was far from monodisperse, but the calculations confirmed the extremely weaker dependence of optical properties on the nanoparticle size and size distribution with respect to that on the concentration, which represents, therefore, the most important parameter here. For both the reference iron oxides and in the whole investigated spectral range, the scattering contribution results were extremely negligible and ω showed a maximum value of 0.0032 for magnetite and 0.0017 for hematite. For this reason, in the rest of this paper, we will refer to extinction and absorption as equivalent words.

**Figure 9:**
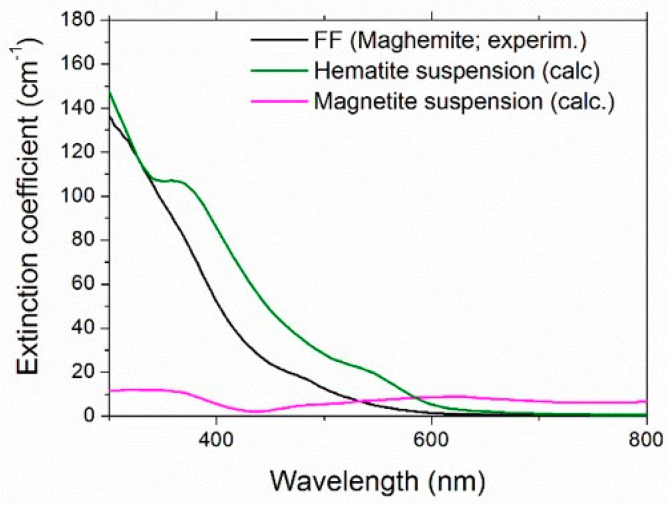
Comparison of experimental extinction coefficient of the maghemite sample and the calculated one for aqueous suspensions of hematite and magnetite.

The extinction efficiency is connected to the extinction coefficient by the relationship [69]:

(16) $$\alpha_{ext}=\rho_{N}\cdot Q_{ext}\cdot \pi a^{2}$$

where *ρ_N_* is the number of nanoparticles per unit volume. Figure 9 compares the experimental extinction coefficient for the maghemite ferrofluid without redox couple and the extinction coefficient calculated for aqueous suspensions of hematite and magnetite nanoparticles at the same concentration. Obviously, as expected, none of the calculated curves reproduced the experimental maghemite one, but hematite was nearer, giving an indirect validation of the previously described scattering estimation, as well as of the choice to approximate the samples as pure optical absorbers.

Looking back at Figure 7, it is possible to see that the ferrofluid was characterized, even at the investigated extremely low nanoparticle concentration, by a very intense absorption for visible wavelengths, where the water base fluid had no absorption bands. The addition of the redox couple further increased absorption by creating at least two broad secondary peaks on the maghemite UV rise front (see the enlargement in Figure 7b). The absorption contribution of nanoparticles and redox couple became negligible after 600 nm. The peaks in the infrared in Figure 7a (centered at around 1500 and 2000, and the incomplete one around 2500 nm) were due to water.

**Figure 10:**
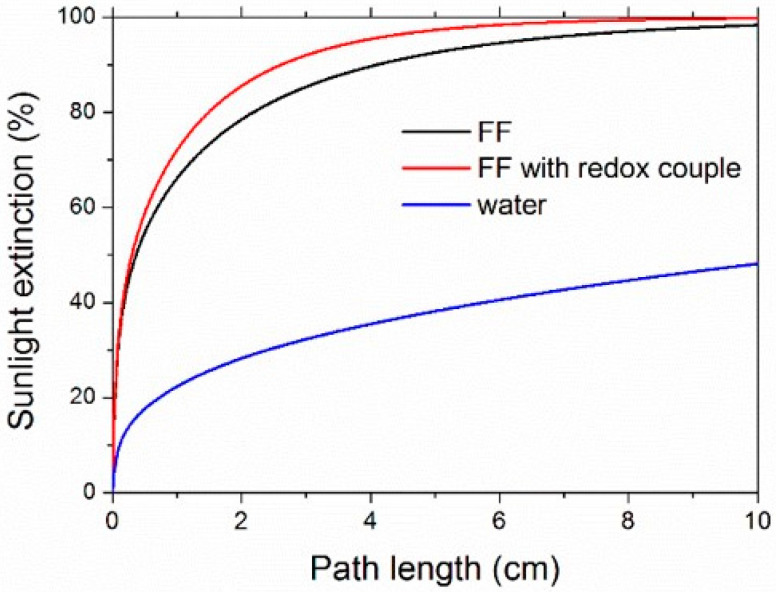
Calculated sunlight extinction as a function of the propagation length within the nanofluids (FF2, Φ = 0.05% vol.).

The sunlight extinction fraction, calculated according to Equation (6), is shown in Figure 10 for ferrofluids and water. Sunlight was absorbed by 90% after a 2.62-cm propagation path in the sample with the redox couple, while the pure ferrofluid needed 4.10 cm for the same level of sunlight absorption. For comparison, water reached about 48% only after a path as long as 10 cm.

As a further comment on Figure 10, it is worth observing that ferrofluid absorption can be easily increased, and the path length giving a desired level of sunlight absorption correspondingly shortened, by increasing the concentration of nanoparticles and/or redox couple [73], allowing us to tailor optical properties according to the geometry of the system where the ferrofluid is employed.

It has to be considered that the presence of nanoparticles and ions in the base fluid also affected the spatial distribution, *S*(*x*), of the stored energy inside the nanofluid volume as a function of the light path length, *x*, which is given by the expression:

(17) $$S\left(x\right)=\frac{\int_{\lambda_{min}}^{\lambda_{MAX}}I\left(\lambda \right)\Delta \mu \left(\lambda \right)\Delta e^{-\mu \left(\lambda \right)x}d\lambda }{\int_{\lambda_{min}}^{\lambda_{MAX}}I\left(\lambda \right)d\lambda }$$

(refer to Equation (6) for the meaning of symbols).

**Figure 11:**
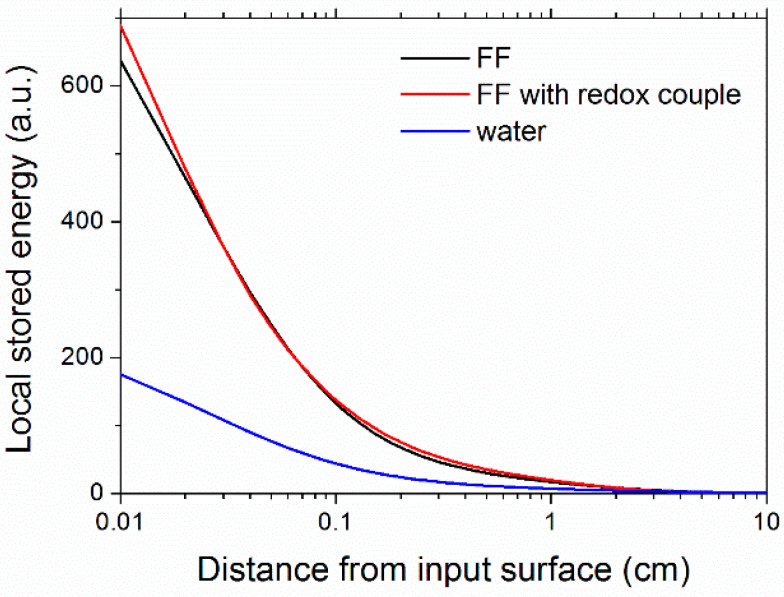
Stored energy distribution in the samples as a function of the distance from the sunlight input surface.

Plots of the calculated stored power distributions along the light propagation direction are reported in Figure 11. Distributions refer to a single-side, irradiated fluid, as in the case of the thickness direction in a flat-plate collector. From Figure 11 it is possible to see that the absorption of ferrofluids was peaked in the first millimeters, where the *visible* part of the input light was absorbed (Figure 7), while inner layers were responsible for the absorption of the *infrared* part of the spectrum.

## 5. Conclusions

In this study, we investigated the thermoelectric and the optical absorption properties of dilute aqueous ferrofluids made with γ-Fe_2_O_3_ magnetic nanoparticles coated by PAAMA molecules. It was found that the inclusion of 0.5% vol. of magnetic NPs accelerates the mass transfer rate of the redox couples within the thermocell when heated from the top, resulting in a 2~3-fold increase of the cell’s power output. The observed enhancement in the mass-transfer rate is suspected to stem from the thermodiffusion (Soret effect) of magnetic NPs, which merits further experimental and theoretical investigations. When the thermocell was heated from below, however, the presence of NPs had little or no effect on its power output, due to the convection becoming the main driving source of mass transfer within the fluid. Therefore, the use of dilute ferrofluids can be advantageous for increasing the thermocell’s efficiency when the device operation imposes the liquid to be heated from the top.

Investigated ferrofluids showed advantageous optical absorption bands in the UV-visible wavelength range, appealing for sunlight absorption. The overall level of optical absorption and its spatial distribution within the nanofluid volume were fully tailorable, acting on the concentration of both magnetic nanoparticles and redox couple ions, allowing the optimization of future sunlight-enabled thermocell geometries.

Together, flat-plate solar thermal collectors, with their large absorption area and ‘hot-over-cold’ heating scheme, are particularly suited for exploiting the optical and thermoelectrical properties of dilute ferrofluids. While we do not yet grasp the mechanism leading to the observed thermoelectric power output increase, additional research in this direction may lead to further improvement toward a new and promising STC concept for the co-generator of heat via volumetric radiation absorption and electricity via thermoelectric generation.

## Figures and Tables

**Table 1:** Comparison of different contributions to the measured internal resistance, *R_i_*(exp), of thermocell under various experimental conditions. The ohmic resistance, *R_O_*, values were determined via AC conductivity measurements while the mass transfer resistance, *R_MT_*, values were theoretically calculated. The charge transfer resistance, *R_CT_*, was taken from literature (see text). Note that *R_MT_* cannot be defined in the cold-over-hot configuration due to the presence of convection.

Φ	*R_i_* (Ω) (exp)	*R_O_*(Ω) (exp)	*R_MT_*(Ω) (calc)	*R_CT_*(Ω) [57]
3 mM redox—Hot over cold configuration				
0	34,000 ± 1000	700	~27,000	<<1
0.5% vol.	12,900± 1000	300	~27,000	<<1
400 mM redox—Hot-over-cold configuration				
0	438 ± 6	9.75 ± 0.5	~200	<<1
0.5% vol.	219 ± 5	9.75 ± 0.5	~200	<<1
400 mM redox—Cold-over-hot configuration				
0	23 ± 1	9.75 ± 0.5	-	<<1
0.5% vol.	23 ± 1	9.75 ± 0.5	-	<<1

## Data Availability

Data will be made available upon reasonable request to the corresponding author.

## Supplementary Materials

The following are available online at https://www.mdpi.com/article/10.3390/nano11041031/s1. Figure S1: (a) Acrylic acid and (b) maleic acid. Figure S2: (a) Homemade thermoelectric cell (also used for AC conductivity measurements). (b) Schematic view of the thermocell operation. ① Platinum electrode in contact with the ferrofluid. ② Copper blocks used to hermetically seal the thermocell and to serve as electric and thermal connections to the electrometer and temperature controller, respectively. ③ Peltier heater/cooler for temperature control. ④ Variable range resistance load for power-output measurements. ⑤ Manual switch. Open circuit = Seebeck coefficient measurements. Closed circuit = power-output measurements.
