# Supplementary material for: Multifunctional Magnetic Nanocolloids for Hybrid Solar-Thermoelectric Energy Harvesting
Source: Nanomaterials (Basel). 2021 Apr 18;11(4):1031. doi: 10.3390/nano11041031 (PMC8074063; doi:10.3390/nano11041031)
Supplement: Supplementary file 1 [file nanomaterials-11-01031-s001.zip › nanomaterials-1176896-supplementary.pdf]

Supplementary Information for:

Multifunctional Magnetic Nanocolloids for Hybrid Solar-Thermoelectric Energy Harvesting

Elisa Sani[1], Maria Raffaella Martina[1], Thomas J. Salez[2,3], Sawako Nakamae[2], Emmanuelle Dubois[4], Véronique Peyre[4]

[1]CNR-INO National Institute of Optics, Largo E. Fermi, 6, I-50125 Firenze, Italy

[2]Service de Physique de l'Etat Condensé, SPEC, CEA, CNRS, Université Paris-Saclay, CEA Saclay, 91191, Gif-sur-Yvette, France

[3]École des Ponts ParisTech, 6 et 8 avenue Blaise Pascal, Champs-sur-Marne, F-77455 Marne-la-Vallée, France

[4]PHENIX, CNRS Sorbonne University, 75005 Paris, France

*corresponding author: elisa.sani@ino.cnr.it

1. PAAMA Ligand

HPAAMA-ligand is a co-polymer composed of acrylic acid and maleic acid (Figure S1(a) and (b)).

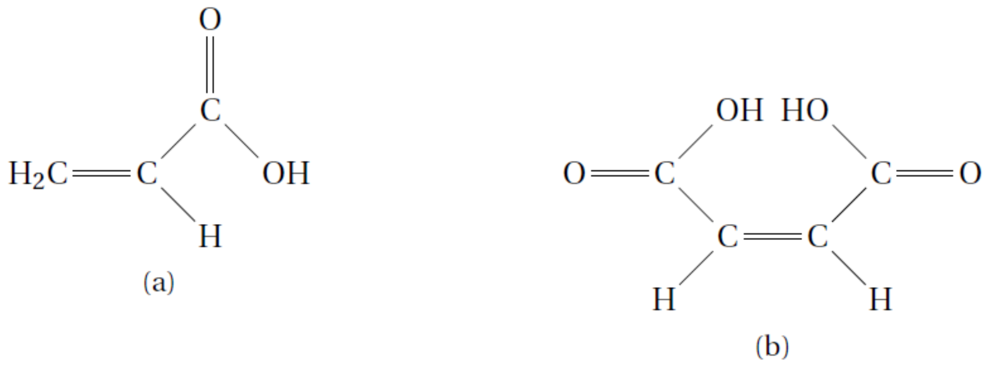

Figure S1 : (a) Acrylic acid and (b) Maleic acid

For the sake of clarity, we will write HPAAMA for the fully protonated (electrically neutral) form while PAAMA will stand for the deprotonated and negatively charged form.

The pKa constant of acrylic acid is 4.25 at 25◦C. For maleic acid, the two pKa at this temperature are 1.92 and 6.23, respectively [1]. At a sufficiently high pH, the carboxylic acid groups are deprotonated and negatively charged, allowing the ionic stabilisation of nanoparticles dispersions. If it is assumed that the pKa of acrylic and maleic acids are not modified too much by the polymerisation, the ionic stabilisation is effective at pH > 2 (the 1st pKa of maleic acid), and is maximum when the pH > 7 (the 2nd pKa of maleic acid). Experimentally, Guibert and co-authors [2] showed that pH > 2.8 is necessary to prevent the nanoparticles aggregation. The ferrofluids used for the present study are in water around pH 7, and thus their stability is well ensured.

2. Synthesis of ferrofluids with PAAMA coating

The ferrofluids with PAAMA-coated nanoparticles is obtained in three successive steps using the so-called precipitation-redispersion procedure [3] [4]; *i.e.*, 1) the replacement of H^+ ligands on the NPs surface by HPAAMA and thus precipitation of NPs, 2) the redispersion of nanoparticles by pH increase and finally, 3) the removal of free (unbound) PAAMA from the ferrofluid.

A 2 wt% aqueous solution of HPAAMA is first prepared from a commercial solution consisting of a statistical co-polymer made of equimolar acrylic and maleic acid monomers (HPAAMA) (Sigma-Aldrich, 50 wt%, average molar mass 3000 g/mol). 25 ml of acid ferrofluid (pH=1.8, in nitric acid) with nanoparticle volume fraction of Φ= 2 vol % is then added dropwise to 50 ml of the bespoke HPAAMA solution under constant stirring to ensure an excess of HPAAMA at all times. This results in a solution composed of NPs coated with HPAAMA. The pH of this solution is ~2.3, lower than the stability threshold of the HPAAMA-coated NPs (see above), therefore the NP precipitation occurs. The solution is placed on a permanent magnet for 30 minutes to allow the flocculation of a maximum number of nanoparticles, creating a biphasic fluid; i.e., an opaque phase with flocculated nanoparticles at the bottom and a transparent supernatant consisting of an aqueous solution of nitric acid. The latter also contains a fraction of nanoparticles that are too small to flocculate even at such a low pH value. After discarding the supernatant, 4 ml of an ammonia solution at pH=12 is added to redisperse the nanoparticles. The dispersion obtained contains a large number of free PAAMA as well as NH^{4+} counter-ions and remaining NO3- ions in addition to the PAAMA coated nanoparticles. In the last step, the free PAAMA ligands and remaining ions (NH4+ and NO3-) are removed by dialysis of the dispersion vs distilled water. The cut-off threshold of the dialysis bag is 10000 Da, which lets the free PAAMA (M=3000 g/mol) and small ions pass through the pores, while retaining the nanoparticles with PAAMA shells. The dialysis is repeated 5 times to ensure the removal of all free PAAMA ligands and ions. The final ferrofluid consists of maghemite nanoparticles coated with PAAMA and therefore negatively charged. The electrical neutrality is ensured by the presence of ammonium ions (counter-ions) in stoichiometric proportion.

3. Ferrofluid characterization (for FF1)

a. MNP volume fraction

The volume fraction was determined from the mass fraction measurements (via UV-visible spectroscopy in a diluted solution (x100)) according to the equation: $\Phi_v=[1+\rho_{NP}/\rho_{H2O}.(1/\Phi_m-1)]^{-1}$ where Φ_v and Φ_m are the volume and mass fractions of NPs. ρ_{NP} is the density of maghemite; 4.88 g/cm^3 and ρ_{H2O}, the density of water.

b. Diffusion coefficient (D) determination

The hydrodynamic radius, R_H of FF1 is deduced from the diffusion coefficients D = 1.80 x 10^{-11} m^2/s, determined using dynamic light scattering technique (DLS), from Stokes-Einstein's law: $R_H=k_BT/(6\pi\eta D)$ with η the dynamic viscosity of the fluid (water). The determined value of R_H is 13.7 nm, reflecting the presence of large PAAMA co-polymer molecules surrounding the NP surface.

c. Interaction energy between magnetic nanoparticles

The magnetic nanoparticles below 10-20 nm in diameter are single-domained, which makes it possible to modeled them, magnetically speaking, as point particles possessing average net dipolar magnetic moment $\vec{\mu}$. The dipolar interaction energy, E_{dd} between two dipole moments $\vec{\mu}_1$ and $\vec{\mu}_2$ separated by $\vec{r}$ is

$$E_{dd} = \frac{1}{r^3}\mu_0[\vec{\mu}_1 \cdot \vec{\mu}_2 - 3(\mu_1\vec{r} \cdot \mu_2\vec{r})]$$

Where μ_0 is the vacuum permeability. E_{dd} is maximum when $\vec{\mu}_1$ and $\vec{\mu}_2$ are collinear. In the case of ~7 nm diameter maghemite particles used in this study, $|\vec{\mu}|$ is of the order of $10^4\mu_B$. (cite which paper?) At the particle concentration of 0.5 %vol., the maximum E_{dd} between two nearest neighboring particles is then calculated using $r^3 = v_{NP}/0.005$ (where v_{NP} is the volume of a single average-sized nanoparticle) to be about .

4. Thermoelectric and ionic conductivity measurements

Thermoelectric (Seebeck coefficient and power-output) measurements were performed in the following manner.

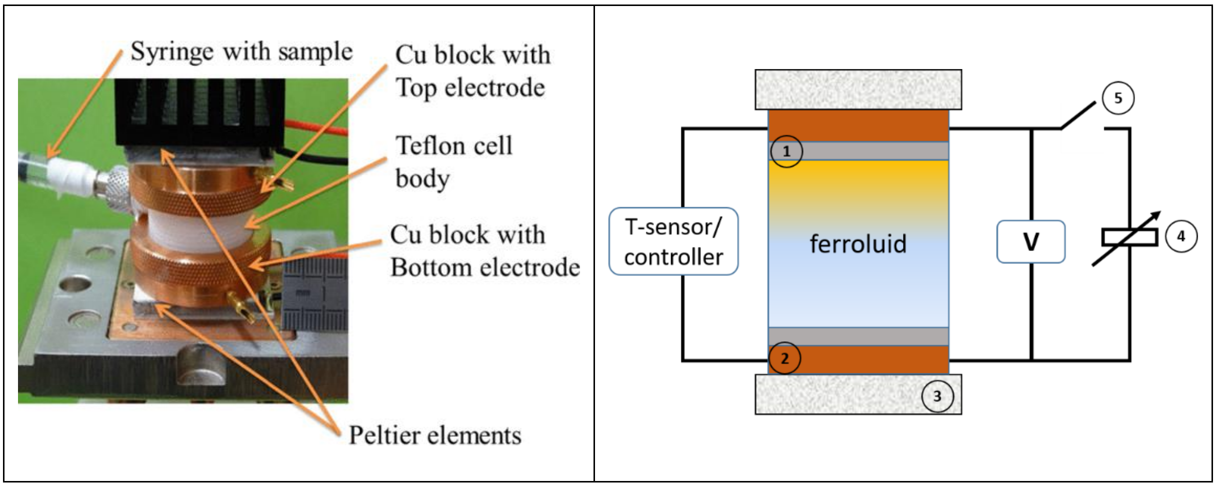

Figure S2 : (a) Home-made thermoelectric cell (also used for AC conductivity measurements). (b) Schematic view of the thermocell operation. ① platinum electrode in contact with the ferrofluid, ② Copper blocks used to hermetically seal the thermocell, and to serve as electric and thermal connections to the electrometer and temperature controller, respectively ③ Peltier heater/cooler for temperature control ④ variable range resistance load for power-output measurements, ⑤ manual switch; open-circuit = Seebeck coefficient measurements, closed-circuit = power-output measurements.

5. Mass-transfer resistance of redox species

Mass transfer resistance of redox species can be estimated from:

$$R_{TM} = max\left(\frac{k_B T \cdot l}{A \cdot n_e^2 \cdot e^2 \cdot D_{ox} c_{ox}}; \frac{k_B T \cdot l}{A \cdot n_e^2 \cdot e^2 \cdot D_{red} c_{red}}\right)$$

Where l and A are the cell length and electrode area, n_e is the number of exchanged electrons in a given reduction-oxidation reaction, $D_{ox/red}$ and $C_{ox/red}$ are the diffusion coefficient and the concentration of oxidizer/reducer species. In this study, where the oxidizing and reducing molecules are of the same size and concentration, the $R_{MT\text{-}ox}$ and $R_{MT\text{-}red}$ are equal.
